# On the impact of vessel wall stiffness on quantitative flow dynamics in a synthetic model of the thoracic aorta

**DOI:** 10.1038/s41598-021-86174-6

**Published:** 2021-03-23

**Authors:** Judith Zimmermann, Michael Loecher, Fikunwa O. Kolawole, Kathrin Bäumler, Kyle Gifford, Seraina A. Dual, Marc Levenston, Alison L. Marsden, Daniel B. Ennis

**Affiliations:** 1 Department of Radiology, Stanford University, Stanford, CA USA; 2 Department of Informatics, Technical University of Munich, Garching, Germany; 3 Division of Radiology, Veterans Affairs Health Care System, Palo Alto, CA USA; 4 Department of Mechanical Engineering, Stanford University, Stanford, CA USA; 5 Department of Bioengineering, Stanford University, Stanford, CA USA; 6 Department of Pediatrics, Stanford University, Stanford, CA USA; 7 Cardiovascular Institute, Stanford University, Stanford, CA USA

**Keywords:** Biomedical engineering, Fluid dynamics, Aortic diseases

## Abstract

Aortic wall stiffening is a predictive marker for morbidity in hypertensive patients. Arterial pulse wave velocity (PWV) correlates with the level of stiffness and can be derived using non-invasive 4D-flow magnetic resonance imaging (MRI). The objectives of this study were twofold: to develop subject-specific thoracic aorta models embedded into an MRI-compatible flow circuit operating under controlled physiological conditions; and to evaluate how a range of aortic wall stiffness impacts 4D-flow-based quantification of hemodynamics, particularly PWV. Three aorta models were 3D-printed using a novel photopolymer material at two compliant and one nearly rigid stiffnesses and characterized via tensile testing. Luminal pressure and 4D-flow MRI data were acquired for each model and cross-sectional net flow, peak velocities, and PWV were measured. In addition, the confounding effect of temporal resolution on all metrics was evaluated. Stiffer models resulted in increased systolic pressures (112, 116, and 133 mmHg), variations in velocity patterns, and increased peak velocities, peak flow rate, and PWV (5.8–7.3 m/s). Lower temporal resolution (20 ms down to 62.5 ms per image frame) impacted estimates of peak velocity and PWV (7.31 down to 4.77 m/s). Using compliant aorta models is essential to produce realistic flow dynamics and conditions that recapitulated in vivo hemodynamics.

## Introduction

Aortic wall stiffness is a strong predictor for all-cause and cardiovascular morbidity in patients with systemic arterial hypertension^1–3^. Model-based studies estimate that a total of 1.56 billion people worldwide may be affected by systemic arterial hypertension by 2025^4,5^. Consequently, monitoring aortic wall stiffness has become increasingly important and could guide treatment strategies and prevention of systemic arterial hypertension. Aortic wall stiffening is linked to an increase of pulse wave velocity (PWV) - the velocity at which the blood pressure pulse travels through the circulatory system. For a vessel of constant diameter, this relationship is modeled by the Moens-Korteweg equation:

1 $$\begin{aligned} \text {PWV} = \sqrt{\frac{\text {E} h}{2 \rho r}} \end{aligned}$$

where E is the elasticity modulus, $\rho$ blood density, *h* wall thickness, and *r* vessel radius. Several PWV measurement technologies exist, with the carotid-femoral PWV (cfPWV) approach considered the clinical gold-standard^6^. cfPWV is approximated using the foot-to-foot temporal shifts of two signal waveforms (e.g. Doppler) recorded transcutaneously at the common carotid and femoral artery. The technical challenges associated with cfPWV measurements, such as carotid-femoral path length measurement inaccuracies or difficulties in transcutaneous signal recording, have limited broader adoption.

Non-invasive 4D-flow magnetic resonance imaging (MRI) provides three-dimensional (3D) and time-resolved velocity vector maps that serve as basis for image-based quantitative flow characterization^7,8^. In particular, 4D-flow PWV calculations use a similar transit-time over fixed distance approach as in conventional cfPWV measurements, but in addition exploits the volumetric imaging data^9^ and analyzes temporal shifts in flow rate waveforms extracted at numerous cross-sectional image planes along the aorta. This approach enables both regionally specific and more robust PWV estimates compared to two-point methods. In addition, the 3D anatomical image information is used to precisely measure the path length. Previous studies suggest low intra- and inter-observer variability and moderate test-retest performance^10,11^. The same studies affirm that PWV increases with age and in the presence of aortic atherosclerosis.

The otherwise lengthy clinical in vivo 4D-flow MRI scan must be accelerated using parallel imaging, compressed sensing, or fast readout techniques^12–17^. Each of these techniques, however, trades-off spatio-temporal image resolution and signal-to-noise ratio (SNR), both of which impact flow quantification accuracy. 4D-flow MRI sampling requirements to report robust PWV values are missing.

In vitro 4D-flow MRI using subject-specific synthetic aorta models connected to a cardiovascular flow pump enables prolonged imaging, thereby allowing optimal image quality. Moreover, in vitro setups enable studying flow dynamics under controllable conditions. In particular, we can program physiological flow waveforms, tune flow volume splits via outlet resistance control, and tune systemic pulse pressure via integration of capacitor elements. The majority of previous studies simplify their setup using rigid wall materials, which neglects the compliant nature of the human vasculature^18–20^. A limited number of studies embed compliant models, but do not report on how the compliance of the model compares to the human aorta^21,22^.

Novel 3D-printing technology permits building models with realistic and varying compliance which we seek to leverage. Herein, this work exploits in vitro 4D-flow MRI with realistic and compliant models of the thoracic aorta to study quantitative flow dynamics. The two objectives of this study were: (1) to demonstrate feasibility of deploying compliant 3D-printed subject-specific aorta models in an MRI-compatible flow circuit setup that matches physiological flow and pressure conditions; and (2) to evaluate the impact of wall stiffness variations on cross-sectional flow metrics and PWV.

## Methods

### Compliant aorta models

**Figure 1:**
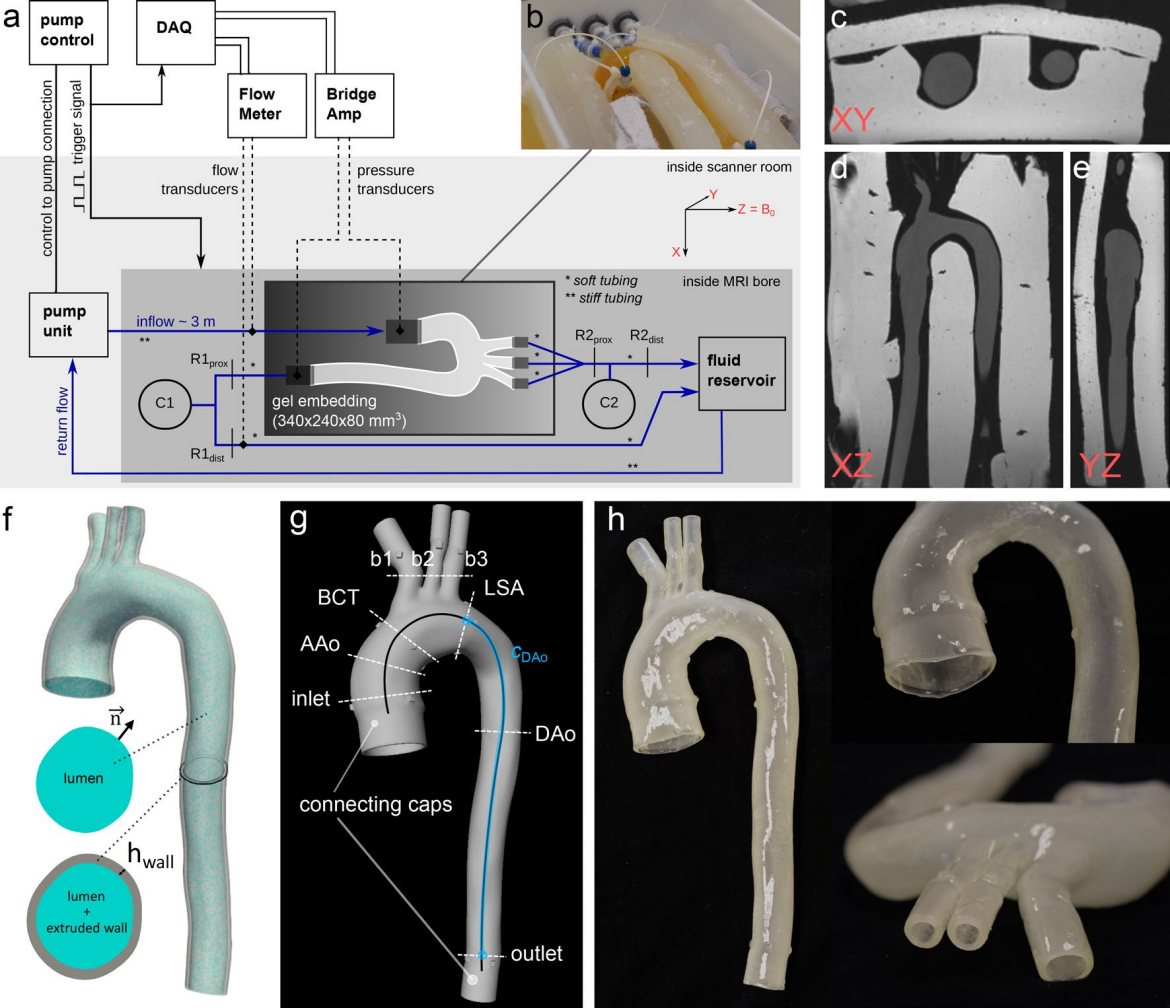
(**a**) Schematic of the MRI-compatible flow circuit setup. The pump unit was positioned at the end of the patient bed; the fluid reservoir was positioned on the patient bed and inside the MRI bore. The pump controller provided a pulse for triggering both image acquisition and DAQ signals. Ultrasonic flow transducers and pressure transducers (dotted lines) were disconnected after tuning and prior to moving the setup to the MRI iso-center. (**b**) Photograph of the model-specific gel block with embedded aorta model and ports (blue) at inlet and outlet to insert pressure transducers. (**c**–**e**) 3D spoiled-gradient echo MRI image data for three reformatted planes (XY, XZ, YZ) depicting the aorta model embedded into the gel. (**f**) Model construction showing lumen mesh (cyan) and extruded wall mesh (gray). (**g**) Final print-ready aortic wall model with defined cross-sectional landmarks, full centerline (black), and descending aorta centerline (blue) that was used for PWV analysis. The original model was extended with cylindrical caps (length = 2 cm) at the inlet and all outlets to accommodate connection to customized barbed connectors that then connect to tubing. (**h**) Photographs of a finished 3D-printed model. Graphics created using Inkscape (v0.92, https://inkscape.org/), SimVascular (release 2020–04, https://simvascular.github.io/), and Meshmixer (v3.5, https://www.meshmixer.com/).

An in vivo chest 4D-flow MRI dataset was acquired from a healthy subject (50 y/o, male) using a protocol that was in accordance with relevant guidelines and regulations, and approved by Stanford University Institutional Review Board. Informed consent was obtained from the subject prior to imaging. The 4D-flow MRI magnitude image was used to generate a subject-specific polygon mesh model of the thoracic aortic wall including the brachiocephalic trunk, left common carotid, and left subclavian artery. The wall domain mesh generation (Fig. 1f) consisted of: (1) binary segmentation of the aortic lumen; (2) polygon surface meshing of lumen mask (edge size = 0.8 mm); (3) extrusion of the mesh nodes in the normal direction to define the outer wall surface mesh; (4) boolean differencing of the outer wall and lumen surface mesh. The resulting wall thickness ($h_{wall}= {2}\, \hbox {mm}$) was within the reported range of the average wall thickness of the human adult aorta^23^. The mesh model was extended by cylindrical caps (length = 20 mm) at the ascending aorta inlet and at the four outlets to enable connection to customized barbed model-tubing transition elements. These steps were performed using SimVascular^24^ and Meshmixer (Autodesk) open software tools.

A photopolymerization 3D printer (J735 PolyJet, Stratasys) with novel printing materials (Agilus30 and VeroClear, Stratasys) was used to manufacture two compliant and one nearly rigid model of the subject-specific aorta geometry (referred to as $\mathrm {M}_{c1}$, $\mathrm {M}_{c2}$, and $\mathrm {M}_{r}$). Printed models were finished with a thin coating (Bectron, Elantas) to prevent fluid absorption (Fig. 1h). To characterize the material stiffness, standardized dumbbell shaped samples (ASTM D412 type A) were 3D-printed in the same batch and using the same material blend as the aorta models.

Uniaxial tensile testing (Instron 5848 Microtester, 10-KN load cell) was performed on three dumbbell samples per material elasticity. Samples were pre-conditioned with five loading and unloading cycles to 10 % peak strain followed by a sixth measurement cycle to 50 % peak strain. Testing was done at ambient conditions with a strain rate of ${25}{\%/\hbox {sec}}$. This rate corresponds to the upper loading rate limit on the aorta models when embedded in the flow circuit, which was assessed by analyzing the dynamic wall circumference in the imaging data. 3D-printing direction anisotropy was evaluated by varying the sample’s orientation on the print bed.

### Flow circuit setup

An MRI-compatible flow circuit setup (Fig. 1a) was engineered to enable *in vitro* 4D-flow imaging of the aorta models under physiological and controllable flow and pressure conditions. The inlets and outlets of the models were sealed to tubing via custom-fit 3D-printed barbed connectors with tapered transitions and then embedded into a ballistics gel block (Fig. 1b, ClearBallistics). The gel block provided a fixed positioning reference and had a short T1 relaxation time, which facilitated using it as static “tissue” for eddy current induced phase offset correction. Aortic model and gel block were placed inside an enclosed box that connects through five box-mounted flow ports to both the pump unit (CardioFlow 5000 MR, Shelley Medical Imaging Technologies) and to the fluid reservoir which supplies the pump unit via a return flow path.

The subject’s previously performed MRI exam included aortic flow measurements from which the flow rate waveform was derived and programmed to the pump unit. To this end, the original waveform was spline-interpolated, down-scaled to meet the pump’s peak flow rate limit (300 mL/s), and discretized ($\Delta t$ = 10 ms) over a cardiac cycle length of RR = 1000 ms (heart rate 60 bpm). The resulting stroke volume was 71.2 mL and total flow was 4.3 L/min.

Flow volume splits across model outlets were controlled via the ratio of clamping the soft downstream tubing (ID = 12.7 mm) at $R1_{dist}$ and $R2_{dist}$ with adjustable pinch valves. We note that $R1_{prox}$ and $R2_{prox}$ were inherently defined by the model-to-capacitor tubing which should be kept as short as possible to most efficiently leverage the effect of the capacitors (described below). Flow splits were assessed using an ultrasonic flow probe (ME-PXL14, Transonic) that clamped-on at the DAo outlet and was connected to a data acquisition system (DAQ) via a flow module (TS410, Transonic). Based on preliminary bench top tests, we targeted a flow volume split of 70/30 for model $\mathrm {M}_{c1}$.

Pulse pressure was controlled using two capacitance elements at the DAo outlet (C1) and at the merged arch branches (C2). The capacitors were designed as cylindrical towers with sealed air compression chambers in which the enclosed air volume (height = 16 cm, diameter = 10.2 cm) dictated the amount of downstream capacitance. Pressure transducers (Micro-Tip SPR-350S, Millar) were inserted at the model inlet and descending aorta outlet, and pressure signal was received at the DAQ through a bridge amplifier front-end (FE224 Quad Bridge, ADInstruments). The mean arterial pressure was elevated by increasing the overall system resistance via the distal pinch valves which—after final tuning—reduced the tubing cross-section to a slit of 1.3–2.3 mm. We defined 110–120 mmHg and 70–80 mmHg as target systolic ($P_{sys}$) and diastolic ($P_{dias}$) pressure ranges for model $\mathrm {M}_{c1}$.

For the three models, identical inflow conditions were programmed, whereas pressure and flow split tuning was performed with model $\mathrm {M}_{c1}$ only. Subsequently, models $\mathrm {M}_{c2}$ and $\mathrm {M}_{r}$ were embedded under the identical periphery without re-tuning system capacitance or resistance. All DAQ data analysis was performed using dedicated DAQ software (LabChart 8, ADInstruments).

### Imaging experiments

Imaging experiments were performed using a 3T MRI scanner (Skyra, Siemens Healthineers) with a 32-channel spine and a 18-channel chest coil. We used a total fluid volume of seven liters (glycerol-water mixture with ratio = 40/60) with $\mathrm {T_1}$-shortening contrast agent (ferumoxytol, concentration = 0.75 mL/L) for increased signal-to-noise ratio (SNR). Protocol steps were as follows: (1) set up “fluid-empty” circuit on the MRI scanner table; (2) flush and de-bubble all fluid lines (steady flow); (3) record and tune pressures and flow splits (pulsatile flow); (4) remove pressure and flow transducers before moving setup to MRI iso-center; (5) run high-resolution 3D spoiled gradient echo (SPGR) acquisition (steady flow, 71.2 mL/s, Fig. 1c–e); (5) run 4D-flow acquisitions (three series, all pulsatile flow); (6) run 2D-PC and 2D-cine-GRE at pre-defined landmark slices (14 series, all pulsatile flow). The pump trigger signal was used for retrospective cardiac gating (direct input to scanner) and to later synchronize signals from pressure and ultrasonic recordings (direct input to DAQ). Image acquisition time per model was 1 hour 45 minutes, and total end-to-end experiment time (including setting up and swapping models) was 10 hours.

#### 2D-PC and 2D-cine-GRE MRI

Two-dimensional (2D) imaging through lumen cross-section was performed at the following landmarks (Fig. 1g): ascending aorta inlet (‘inlet’), ascending aorta (‘AAo’), arch just proximal to brachiocephalic trunk (‘BCT’), and distal to left subclavian artery (‘LSA’), mid-descending aorta (‘DAo’), descending aorta outlet (‘outlet’), and three arch branches (‘b1’, ‘b2’, ‘b3’). 2D MRI sequences included: (1) 2D cine gradient echo (2D-cine-GRE) with in-plane resolution 0.9 $\times$ 0.9 mm, slice thickness = 6 mm, FoV = 240 $\times$ 150 mm, TE/TR = 3/4.75 ms, flip angle = 7$^{\circ }$, averages = 2, retrospective gating, number of temporal frames = 50 (frame length = 20 ms), no parallel imaging acceleration; and (2) 2D phase-contrast (2D-PC) with $V_{enc}$ = 90–120 cm/s, in-plane resolution 1.1 $\times$ 1.1 mm, slice thickness = 6 mm, FoV = 220 $\times$ 123 mm, TE/TR = 3/21 ms, flip angle = 25$^\circ$, averages = 2, retrospective gating, number of temporal frames = 50 (frame length = 20 ms), no parallel imaging acceleration.

#### 4D-flow MRI

We used a conventional 4D-flow sequence with Cartesian k-space sampling, a velocity encoding range ($V_{enc}$) of 120 cm/s, and repeated scans at three temporal resolutions (20 ms, 40 ms, 62.5 ms), leading to a total of nine datasets (Table 1). $V_{enc}$ was chosen to optimize signal-to-noise ratio during systole while avoiding phase-wrapping artifacts, i.e. just above peak systolic velocities as measured by preceding 2D-PC. To minimize phase offsets and to improve geometric fidelity, the image data were corrected for Maxwell terms (during reconstruction), gradient non-linearity, and eddy current (both post-reconstruction). Distortion correction due to gradient non-linearity was implemented as described by Markl et al.^25^ 3D phase images were corrected for eddy current effects via linear fitting of 3D offset maps through the ballistics gel image region. No phase unwrapping was required. 4D-flow images were processed using MEVISFlow software solution (v11.2, Fraunhofer Institute for Digital Medicine)^26^.

**Table 1:** 4D-flow MRI sequence parameters.

	High temp-res	Baseline temp-res	Low temp-res
FoV ($\mathrm {mm}^3$)	$360\times 260\times 100$	$360\times 260 \times 100$	$360\times 260\times 100$
Acquisition matrix	$144\times 104\times 40$	$144\times 104\times 40$	$144\times 104\times 40$
Spatial resolution (mm)	2.5 isotropic	2.5 isotropic	2.5 isotropic
Lines per segment	1	2	3
Reconstructed frames	50	25	16
Temporal resolution (ms)	20	40	62.5
TE/TR (ms)	2.8/5.2	2.8/5.2	2.8/5.2
$V_{enc}$ (cm/s)	120	120	120
Flip angle ($\circ$)	15	15	15
BW (Hz/px)	451	451	451
Scan time (mm:ss)	42:40	21:20	14:40

Three data sets were acquired with each model, resulting in a total of nine datasets available for analysis. Variations in effective temporal resolution were controlled by the number of acquired k-space lines per segment, and the number of reconstructed cardiac frames was adapted accordingly. *FOV* field of view, *TE* echo time, *TR* repetition time, $V_{enc}$ velocity encoding range, *BW* bandwidth.

### Image analysis

#### 2D-PC and 2D-cine-GRE analysis

For all cross-sectional landmarks (Fig. 1g) aorta lumen contours were manually drawn in the first cardiac frame ($t = 0$) of the 2D-cine-GRE slices and tracked through all subsequent frames ($t = 1{-}49$) using a phase-based motion tracking algorithm as described by Tautz et al.^27^. Lumen expansion was assessed by calculating relative contour area change over time. Identical contours were used to assess inflow conditions and net flow splits based on the acquired 2D-PC data.

#### 4D-flow analysis

For each aorta model, we segmented a 3D aorta lumen mask $m[{\varvec{x}}]$ in the 3D SPGR image data using an automated 3D region growing algorithm, and subsequently derived the lumen centerline ($c_{full}$) using a skeleton approach^28^ on the 3D binary mask. The centerline was used to define cross-sectional planes for 4D-flow parameter quantification at landmarks identical to those defined as part of the 2D acquisitions (Fig. 1b) and to extract equidistant flow waveforms used for the PWV computation.

Based on the 4D-flow magnitude image output, time-resolved lumen contours were automatically tracked as described above. The following 4D-flow based metrics were computed: flow rate (mL/s), net flow per cycle (mL), and mean/max velocity (cm/s). All metrics were compared for all combinations of model wall stiffness and temporal resolution.

#### PWV calculation

PWV computation was focused on the descending aorta only (with centerline $c_{DAo}$, reaching from landmarks ‘LSA’ to ‘outlet’), owing to flow effects at the arch branches that alter flow waveforms and compound the computation. Given the full velocity vector field ${\varvec{v}}[{\varvec{x}},t]$, lumen mask $m[{\varvec{x}}]$, and $c_{DAo}$, flow rate curves were computed as follows: (1) define N cross-sectional analysis planes with normal vector ${\varvec{n}}_k$ with $k = [1,\mathrm {N}]$ at equidistant points $c_k$ along $c_{DAo}$ (spacing = 5 mm) ; (2) retrieve oriented lumen cross-sections $A_k$ at planes defined by ${\varvec{n}}_k$, $c_k$, and lumen mask $m[{\varvec{x}}]$; (3) compute flow rate

$$\begin{aligned} Q_k [t] = \int \langle {\varvec{v}}[{\varvec{x}},t], {\varvec{n}}_k\rangle d{A_k}. \end{aligned}$$

$Q_k[t]$ curves were interpolated using cubic-splines and the time-to-foot (TTF) approach (Fig. 7a) was used to calculate PWV^29^. Briefly, $\mathrm {TTF}_k$ for each $Q_k[t]$ curve was defined as the x-intercept of a line fitted through the waveform’s upslope points at 20 % and 80 % of the peak flow rate. $\mathrm {TTF}_k[c_k]$ was plotted as function of the centerline location and ${\varvec{\mathrm {PWV}_{TTF}}}$ was defined as the inverse slope of the linear regression line fitted to $\mathrm {TTF}_k[c_k]$. Linear regression used a conventional least-square-error (LSE) approach as well as a random sampling consensus (RANSAC) algorithm to better handle outlier.

## Results

### Tensile testing

All material samples exhibited non-linear stress-strain behavior. The incremental Young’s moduli were estimated by the tangent modulus ($E_{t}$) at nominal stress $\sigma ={0.053} \hbox {MPa}$, which was approximated by $\sigma = Pr/h_{wall}$ with *P* given by the recorded mean pressure $P_{MAP} = {57} \hbox {mmHg}$ (during pulsatile flow), wall thickness $h_{wall} = {0.002} \hbox {m}$, and average lumen radius $r = {0.014} \hbox {m}$. $E_{t}$ for the compliant models $\mathrm {M}_{c1}$ and $\mathrm {M}_{c2}$ were 1.27MPa (ranging 1.23MPa to 1.31MPa) and 4.3MPa (ranging 3.7MPa to 4.88MPa), respectively. As for model $\mathrm {M}_{r}$, no absolute elasticity estimates could be derived from the given stress-strain data, but differences in $\mathrm {M}_{r}$ elasticity were approximated to be at least 15-fold $({>15} \hbox {MPa})$ when compared to model $\mathrm {M}_{c1}$. 3D-printing anisotropy was negligible with differences ${<5 \%}$ within the relevant strain range for all three samples. Stress-strain plots are shown in Supplementary Fig. S1.

### Pressure tuning

**Figure 2:**
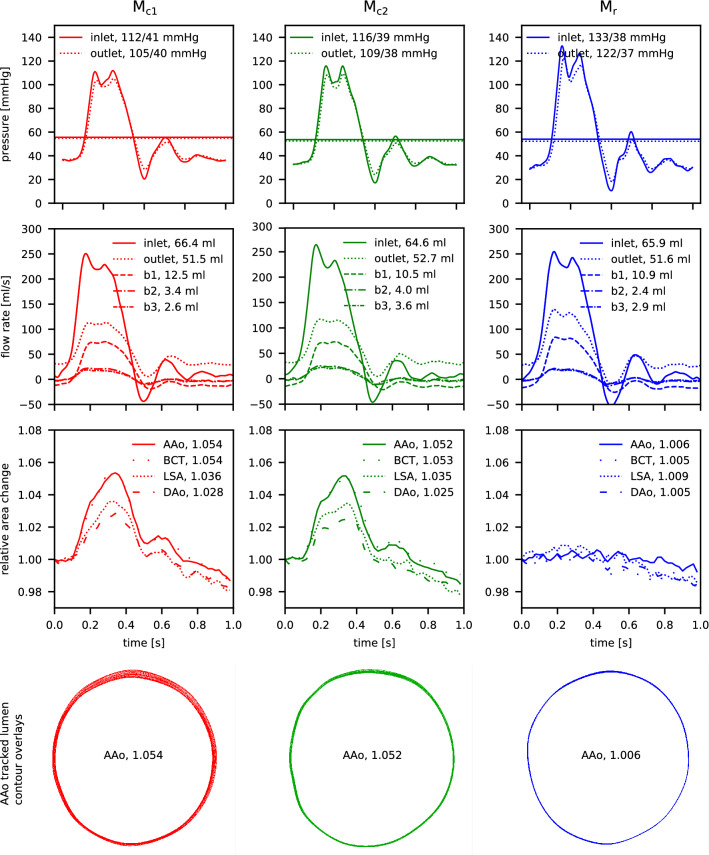
Experimental setup conditions for aorta models $\mathrm {M}_{c1}$ (red), $\mathrm {M}_{c2}$ (green), and $\mathrm {M}_{r}$ (blue), evaluated at selected landmarks (Fig. 1g). (Row 1) Pressure conditions recorded at inlet (solid) and outlet (dashed). (Row 2) Flow rate waveforms at inlet and all outlets with calculated net flow volumes, based on 2D PC-MRI data. (Row 3) Cross-sectional area change relative to area at cardiac cycle start, based on tracked lumen contours in 2D-cine-GRE data. (Row 4) Overlay of tracked lumen contour at cross-section AAo for all acquired time frames (N = 50). Animated contour tracking results are presented in Supplementary Fig. S2. Plots created using Python (v3.6, https://www.python.org/).

Pressure recordings were performed on the scanner bed prior to image acquisition. Pressure waveforms (Fig. 2, top row) recorded at the model inlet show increased peak pressures in models $\mathrm {M}_{c2}$ (116mmHg) and $\mathrm {M}_{r}$ (133mmHg) compared to the most compliant model $\mathrm {M}_{c1}$ (112mmHg). At the model outlet (DAo branch) peak pressures dropped by 7mmHg for both compliant models $\mathrm {M}_{c1}$ and $\mathrm {M}_{c2}$, and by 11mmHg for the nearly rigid model $\mathrm {M}_{r}$. Diastolic pressure values were between 38mmHg and 40mmHg for all models at the inlet and dropped by 1mmHg at the outlet. All pressure waveforms showed an oscillating behaviour both in systole and diastole, which was most dominant in model $\mathrm {M}_{r}$.

### Flow split tuning

Flow splits between all model outlets were consistent between the three models, with net flows of 48–49 ml measured with the ultrasonic probe at the descending aorta outlet prior to each acquisition (corresponding 68% of the programmed inlet flow). Inlet net flow volumes calculated from 2D-PC flow rate waveforms (Fig. 2, row 2) ranged from 64.6 to 66.4 ml; 2D-PC DAo outlet net flow volumes ranged from 51.6 to 52.7 ml. Adding up 2D-PC measured net flow at all outlets (b1, b2, b3 and DAo) total outflow was 70.0, 70.8, 67.8 ml for $\mathrm {M}_{c1}$, $\mathrm {M}_{c2}$, and $\mathrm {M}_{r}$, corresponding to relative differences of 1.7, 0.6, and 4.8 % from the programmed inflow (71.2 ml).

### Aorta wall expansion

Aortic wall expansion was clearly visible in systole for models $\mathrm {M}_{c1}$ and $\mathrm {M}_{c2}$ and most pronounced in the ascending aorta. The wall expanded non-uniformly for all evaluated landmarks (Fig. 2, row 4, and Supplementary Fig. S2) owing to the posterior constraint provided by the gel block (Fig. 1c). Based on the tracked contours, the calculated cross-sectional area increased by ${>5}\%$ in models $\mathrm {M}_{c1}$ and $\mathrm {M}_{c2}$, whereas no detectable area change $({<1}\%)$ was measured for model $\mathrm {M}_{r}$ (Fig. 2, row 3). For models $\mathrm {M}_{c1}$ and $\mathrm {M}_{c2}$, relative area change over the cardiac cycle also depicted a small secondary lobe in early diastole which was in phase with the secondary lobes of the pressure and flow rate waveforms.

### Velocities at cross-sections

**Figure 3:**
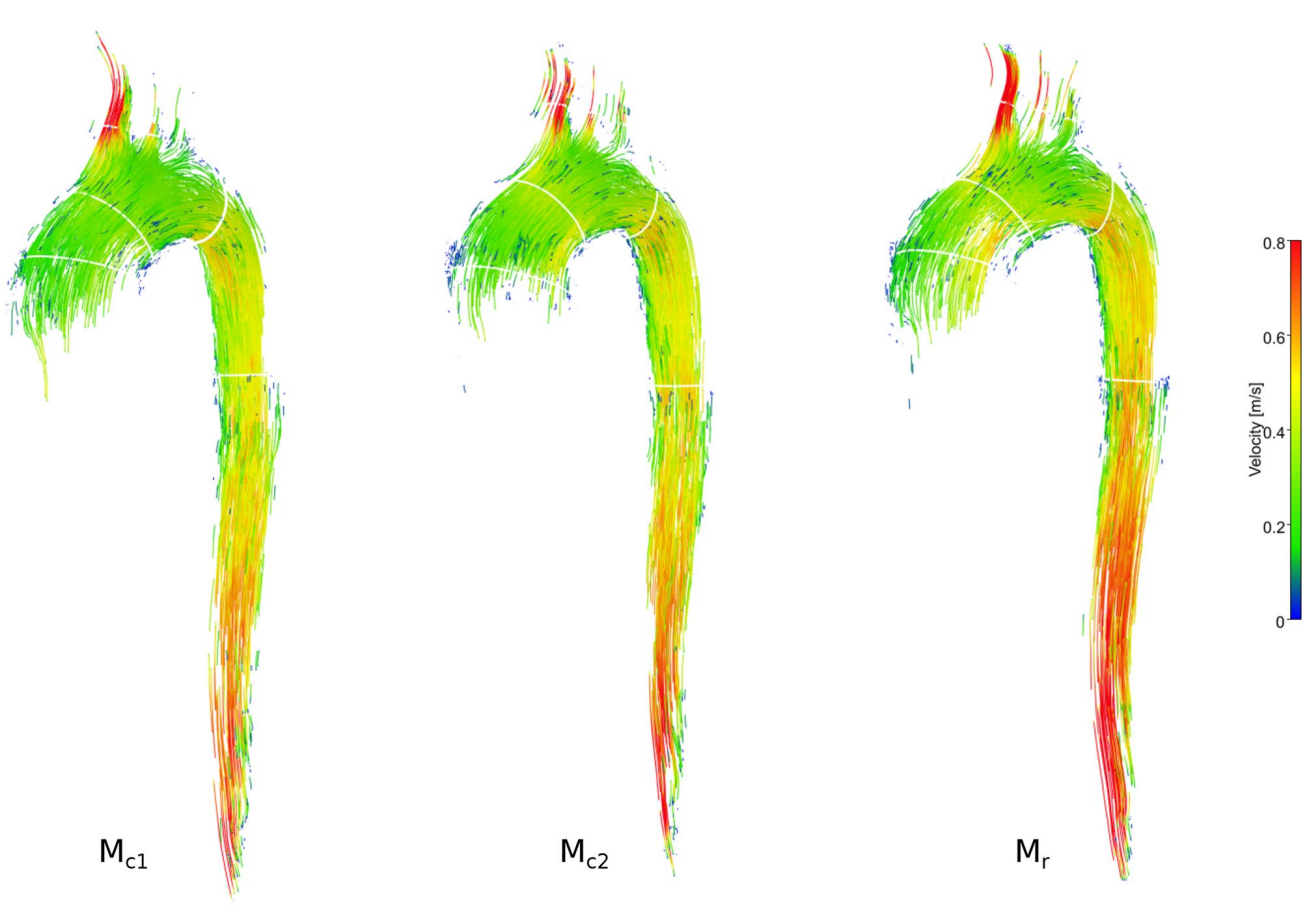
Particle tracing based on 4D-flow MRI data for the three aorta models of identical subject-specific geometry, but different wall stiffness. While particle traces matched among the three models, velocities along the descending aorta—as depicted by the color—were slightly higher in the nearly rigid model $\mathrm {M}_r$. An animated version of traced particles is shown in Supplementary Video S3–5. Graphic created using MevisLab (v3.4a, https://www.mevislab.de/).

**Figure 4:**
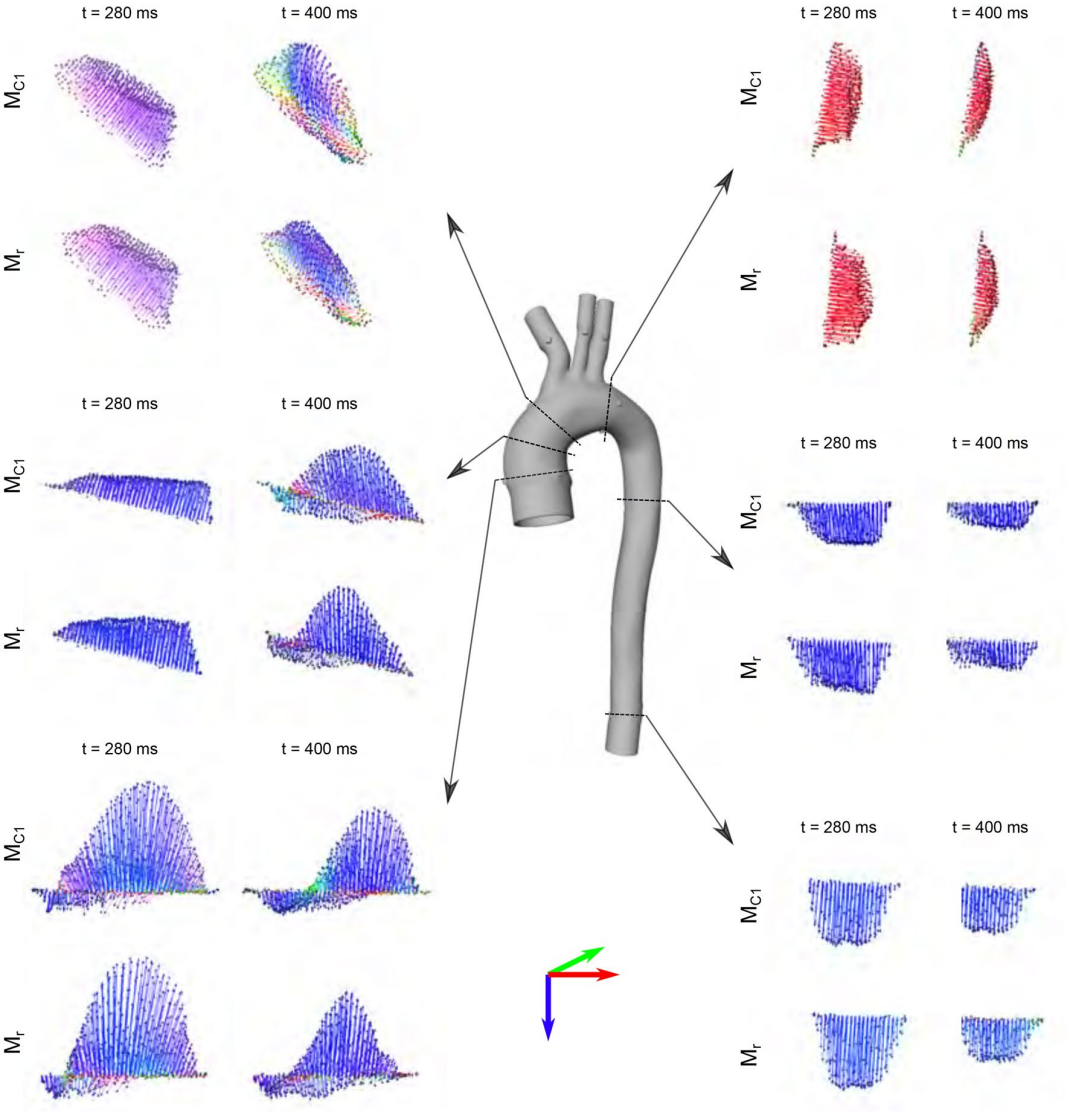
4D-flow cross-sectional velocity profiles in models $\mathrm {M}_{c1}$ (top) and $\mathrm {M}_{r}$ (bottom) at peak-systolic ($t = {280}\,\hbox {ms}$) and end-systolic ($t = {400} \,\hbox {ms}$) frames. All profiles are 3D-rendered using the identical camera view and colored according to the 3D direction (red-green-blue arrow legend). Backward flow is visible at cross-sections prior to the arch branches (inlet, AAo, BCT), specifically at end-systole. One can appreciate the different vector profiles for the compliant model $\mathrm {M}_{c1}$ when compared to the nearly rigid model $\mathrm {M}_{r}$. Velocity profiles rendered using MevisLab (v3.4a, https://www.mevislab.de/).

Figure 4 shows a qualitative comparison between model $\mathrm {M}_{c1}$ and model $\mathrm {M}_{r}$ and their velocity vector profiles for cardiac frames at peak systole and end systole. While profiles in models $\mathrm {M}_{c1}$ and $\mathrm {M}_{r}$ were similar at peak systole, minor qualitative differences were observed at end systole, particularly at landmarks proximal to the arch branches. Here, the nearly rigid model $\mathrm {M}_{r}$ showed a more centered cross-sectional velocity profile with less backward flow components and less helical flow tendencies. Velocity profiles at landmarks distal to the arch branches mainly differed with regards to the velocity vector magnitude, with higher velocities in model $\mathrm {M}_{r}$. Model dependent velocity differences in the descending aorta can also be observed in maps of traced particles, as visualized in Fig. 3.

**Figure 5:**
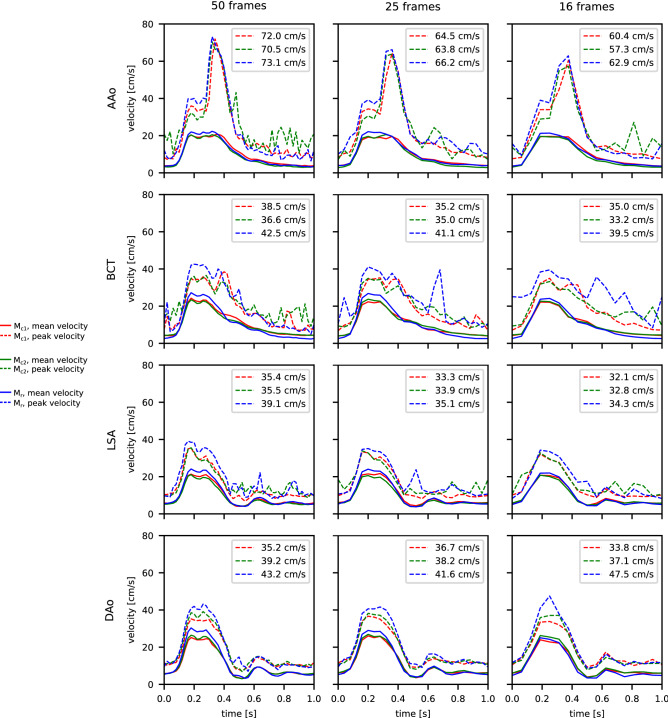
4D-flow cross-sectional mean (solid) and maximum (dashed) velocity at four landmarks (Fig. 1g) and three different temporal sampling rates. Highest peak-systolic velocities (see values in legends) were measured in model $\mathrm {M}_{r}$ (blue); and inter-model peak-systolic velocity differences were greater at landmarks further downstream. The temporal sampling rate impacted the measurement of peak velocities, which was most pronounced at AAo point. Spikes in diastole were attributed to noise in near-boundary pixels and inaccurate contouring. Plots created using Python (v3.6, https://www.python.org/).

Figure 5 shows mean and maximum velocity analysis results. Maximum velocities at peak systole were highest in model $\mathrm {M}_{r}$ (73.1 cm/s at AAo, 42.5 cm/s at BCT, 39.1 cm/s at LSA, and 43.2 cm/s at DAo) compared to both compliant models $\mathrm {M}_{c1}$ (73.0 cm/s at AAo, 38.5 cm/s at BCT, 35.4 cm/s at LSA, and 35.5 cm/s at DAo) and $\mathrm {M}_{c2}$ (70.5 cm/s at AAo, 36.6 cm/s at BCT, 35.5 cm/s at LSA, and 39.2 cm/s at DAo). Likewise, cross-sectional mean velocities in systole were higher in model than in the compliant models. Decreasing temporal sampling from 50 frames down to 16 frames showed greatest effects at the AAo landmark for peak velocity which decreased by 16%, 19 %, 14 % for $\mathrm {M}_{c1}$, $\mathrm {M}_{c2}$, and $\mathrm {M}_{r}$.

### Flow at cross-sections

**Figure 6:**
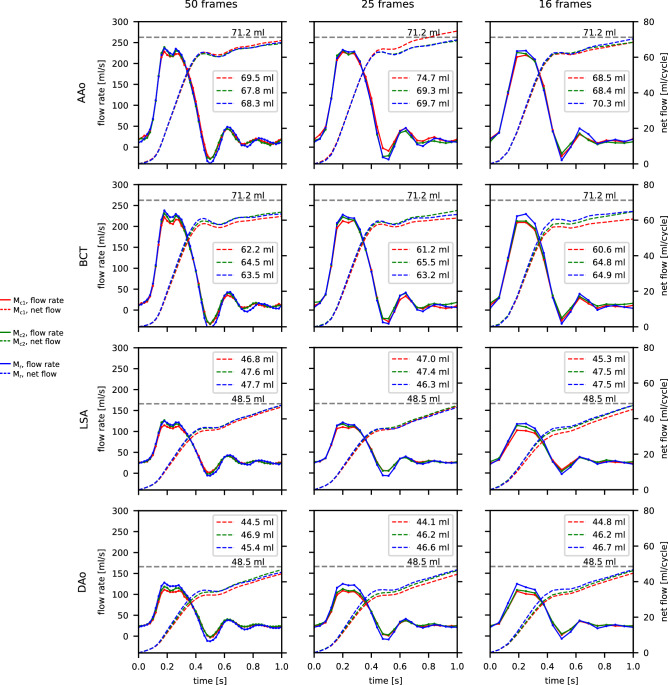
4D-flow derived flow rate waveforms (left ordinates) and cumulative net flow (right ordinates) at four landmarks (Fig. 1g) and three different temporal sampling rates. Grey horizontal lines show the expected net flow according to the programmed inflow (71.2 mL/cycle) for the AAo and BCT slices, and measured (via ultrasonic transducer during setup tuning) DAo branch outflow (48.5 mL/cycle) for the LSA and DAo slices. Plots created using Python (v3.6, https://www.python.org/).

Figure 6 shows flow rate and cumulative net flow results. Results were similar between different models or between 4D-flow data of different temporal sampling rates. Net flow values were $69.6\pm 1.9\, \mathrm {ml}$ for AAo, $63.4\pm 1.6\, \mathrm {ml}$ for BCT, $47.0\pm 0.7\,\mathrm {ml}$ for LSA, and $45.7\pm 1.0\, \mathrm {ml}$ for DAo (given as $\mathrm {mean}\pm \mathrm {SD}$ over three models and three temporal sampling rates). Net flow at the landmarks upstream and downstream of the arch branches were within 10% of the programmed pump value (71.2 ml) and the measured ultrasonic value (48.5 ml), respectively.

Flow rate waveforms over the cardiac cycle (Fig. 6, solid lines) showed weaker peak flow rate dampening with increased model wall stiffness. This effect was most pronounced at landmarks further downstream (LSA, DAo). 4D-flow sampled at 50 frames per cycle revealed a double flow rate peak in systole at all cross-sections, which was much less apparent in the dataset sampled at 25 frames per cycle and not apparent in the dataset sampled at 16 frames per cycle. Moreover, a distinct second (t = 0.66 s) and third (t = 0.85 ms) flow rate peak were present in all derived waveforms, irrespective of model stiffness and temporal sampling rate.

### PWV

**Figure 7:**
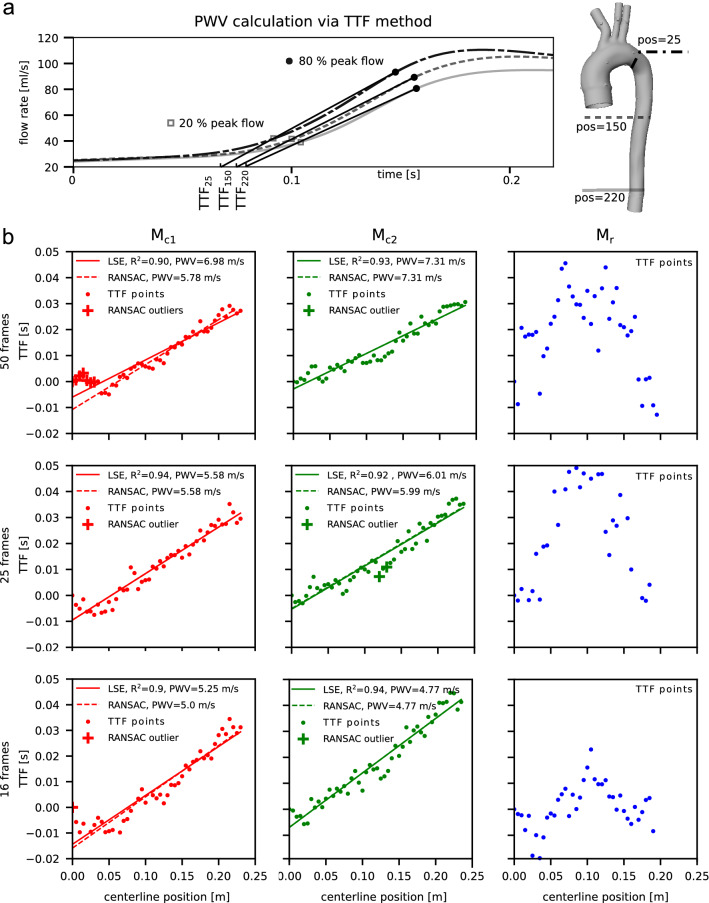
(**a**) Principle of retrieving TTF values at three positions along the descending aorta centerline. (**b**) PWV calculations for models $\mathrm {M}_{c1}$ (red), $\mathrm {M}_{c2}$ (green), and $\mathrm {M}_{r}$ (blue) for three temporal sampling rates (row 1 through 3). Scattered points depict TTF of flow rate waveforms extracted at equidistantly spaced cross sections along $c_{DAo}$ (Fig. 1f). All TTF values are shown as TTF differences to TTF at centerline position 0. Conventional LSE linear regression (solid line, with $R^2$ given in legend) and RANSAC (dashed line with assigned outlier marked +) were used to derive PWV values, defined as the inverse slope of the respective line (given in legend). Differences in PWV were observed between models $\mathrm {M}_{c1}$ and $\mathrm {M}_{c2}$. However, temporal sampling rates impacted these values, with decreasing PWV estimates for lower temporal resolution. It appears that PWV in model $\mathrm {M}_{r}$ is too fast such that plausible TTF along the centerline cannot be resolved with the 4D-flow based approach. LSE is very sensitive to outlier data which was apparent in data $\mathrm {M}_{c1}$ (50 frames) and $\mathrm {M}_{c1}$ (16 frames). For the latter one, four TTF points laid outside the displayed y-axis with linear regression deviations >100 $\times$ RMSE and were thus excluded prior to fitting the model. TTF, time-to-foot; LSE, least-squared-error; RANSAC, random sampling consensus; RMSE, root-mean-squared-error. Plots created using Python (v3.6, https://www.python.org/).

PWV values were estimated from time-to-foot (TTF) delays of flow rate waveforms at equidistantly spaced cross-sectional planes along the descending aorta (Fig. 7b). Based on the datasets with highest temporal sampling (50 frames/cycle), PWV was 6.98 m/s (LSE) and 5.78 m/s (RANSAC) for model $\mathrm {M}_{c1}$, and 7.31 m/s (LSE) and 7.31 m/s (RANSAC) for model $\mathrm {M}_{c2}$. Both for model $\mathrm {M}_{c1}$ and $\mathrm {M}_{c2}$, PWV values were lower with datasets sampled at 25 frames/cycle and further decreased for datasets sampled at 16 frames/cycle. The 16 frames/cycle dataset of model $\mathrm {M}_{c1}$ included four extreme outlier points posting large negative TTF values. These points were excluded prior to fitting the linear model. No linear relationship between TTF and centerline position was detectable for model $\mathrm {M}_{r}$ irrespective of temporal resolution. Consequently, while PWV was very high, no PWV values could be reported based on the data.

## Discussion

This study demonstrated the feasibility of integrating a subject-specific aorta model with varying wall elasticity into an MRI-compatible flow circuit setup that operates under physiological flow and pressure conditions. Utilizing prolonged and highly-controlled in vitro 4D-flow imaging, we showed the influences of aortic wall compliance and temporal sampling rates on both cross-sectional flow parameters and 4D-flow derived PWV.

Stress-strain testing of the compliant 3D-printing material suggested that the derived tangent moduli $E_{t}$ of models $\mathrm {M}_{c1}$ and $\mathrm {M}_{c2}$ are in the same range as the incremental Young’s moduli that have been reported for a ‘young’ (more compliant) and ‘old’ (stiffer) human thoracic aorta, respectively^30^. We did not attempt to report $E_{t}$ for the non-realistic model $\mathrm {M}_{r}$ due to the material’s substantially higher stiffness which challenged reliable tensile testing, but approximated the difference in elasticity to be at least 15-fold when compared to model $\mathrm {M}_{c1}$.

Pressure tuning was performed for model $\mathrm {M}_{c1}$ only. Subsequently, model $\mathrm {M}_{c1}$ was interchanged with models $\mathrm {M}_{c2}$ and $\mathrm {M}_{r}$, but resistance and downstream capacitance were kept constant. This approach allowed for isolated evaluation of the effect increased wall compliance on pressure and flow. While tuning model $\mathrm {M}_{c1}$ to physiological $P_{sys}$ was successful, $P_{dias}$ was below the target range of 70–80 mmHg. Previous work with advanced MRI-compatible flow circuit setups reported similar increased (i.e. ${>50}\hbox {mmHg}$) pulse pressures^21,31,32^. We note that two main factors determine successful pressure tuning: (1) $P_{sys}$ or $P_{MAP}$ can easily be elevated by increasing flow resistance distal to the capacitors for which the pulse pressure remains constant; (2) pulse pressure is governed by the available capacitance, i.e. compressible air volume (C1, C2) and by the ratio of distal to proximal resistance ($R1_{dist}/R1_{prox}$, $R2_{dist}/R2_{prox}$). A greater ratio provides a wider range for tuning pulse pressure. Despite $P_{dias}$ being lower than physiological values, the achieved conditions were found to be sufficient to study the impact wall compliance on flow dynamics. Interchanging models under consistent resistance and capacitance settings led to two effects on pressure: (1) $P_{sys}$ increased in model $\mathrm {M}_{c2}$, and more so in model $\mathrm {M}_{r}$, but no effects were seen on $P_{dias}$; (2) inlet to outlet peak pressure differences increased with increasing wall stiffness. Both of these observations were as expected and they affirmed the validity of the setup.

Pressure and flow rate oscillations (Fig. 2) are expected to be caused by wave reflections at several branching points—natural arch branches, rigid flow connectors, flow valves, etc.—and under-damping in the system. Other studies with comparable flow circuit setups showed similar oscillating waveform shapes^21,31,32^. Additional engineering efforts to mitigate this phenomenon may benefit analyses of pressure and flow waveform shapes in multiple vessel geometries and/or under varying boundary conditions.

4D-flow image-based visualizations of vector profiles and traced particles indicated that variations in wall compliance lead to variations in velocity amplitudes and profiles (Figs. 3, 4). The quantitative analyses showed that both mean and maximum velocities decreased for the compliant models when compared to the nearly rigid version. Likewise, flow rate waveform dampening was most pronounced in the most compliant model and the further downstream the centerline. Thus, in comparative in vitro to in vivo studies—regardless of efforts to match patient-specific inflow conditions—fully rigid aorta models are likely insufficient for direct comparison.

4D-flow based PWV calculations in compliant models $\mathrm {M}_{c1}$ and $\mathrm {M}_{c2}$ provided values within the range of PWV values that have been reported in in vivo 4D-flow studies^11,33,34^. PWV in the model $\mathrm {M}_{r}$ was too high to reliably resolve TTF delays along the centerline. Thus, we did not attempt to report a 4D-flow derived PWV for model $\mathrm {M}_{r}$. The referenced *in vivo* studies included healthy volunteers (young and old) and patients with atherosclerosis. Mean PWV among the respective cohorts ranged from 3.8 to 6.4 m/s. However, they did not include PWV measurements based on multiple 4D-flow datasets with varying temporal resolutions, which ranged from 32–41 ms—a typical temporal resolution in in vivo 4D-flow acquisitions amid scan time limitations. Another previous study utilizing through-plane encoded 2D-PC MRI with higher temporal resolution (6–10 ms), reported PWV values ranging from 4.3 m/s (healthy and young controls) to 6.5 m/s (older patients).

In contrast to the reported in vivo 4D-flow based PWV values, theoretical PWV values based on Moens-Korteweg (Eq. 1) are 8–10.7 m/s for model $\mathrm {M}_{c1}$, 14.9–20 m/s for model $\mathrm {M}_{c2}$, and 31–41 m/s (assuming $E_t$ of model $\mathrm {M}_{r}$ to be 15-fold over model $\mathrm {M}_{c1}$). The given ranges correspond to the change of aortic diameter, which ranges from 36 mm in the ascending aorta to 20 mm in the distal descending aorta. These theoretical values may be debated, as the Moens-Korteweg equations assumes a constant vessel diameter, which is not true of the aorta.

Variations in 4D-flow temporal resolutions affected PWV considerably. Assuming that the presented 4D-flow data at highest temporal resolution ($\Delta {t} = {20}\,\hbox {ms}$) generates the most reliable PWV values, the present results suggest that lower temporal sampling rates underestimate absolute PWV (up to 35 %). Specifically, our data shows that the impact of temporal resolution on PWV calculation may be more dominant than the effect of varying wall compliance. One TTF plot (model $\mathrm {M}_{c1}$, 16 frames) included distinct outlier points with negative TTF delay that were removed prior to linear model fitting. This emphasizes that reliable PWV calculations are highly dependent on accurate flow waveforms, particularly when derived from data with low temporal resolution. In that case, using the alternative iterative RANSAC approach for fitting a linear regression showed the effect on PWV while directly excluding these outlier points.

Four key limitations of this study were identified. First, only a single approach for PWV measurement (TTF) was used. In addition to the TTF, others derived PWV by time-to-peak (TTP), time-to-upstroke (TTU), and by correlation analysis of time-shifted flow waveforms (xCorr)^29,34,35^. Wentland et al.^34^ analyzed differences in PWV for these four approaches. PWV values were similar for TTF, TTU and xCorr, while TTP results deviated most due to challenges of detecting the true peak flow point in data with mediocre temporal resolution.

Second, synthetic aorta models were manufactured with uniform wall thickness and elasticity which simplifies the in vivo aorta. These local variations of the model may impact calculated PWV values. A subject-specific wall mesh directly built on a vessel wall segmentation—rather than segmenting the lumen and extruding the surface by a pre-defined and uniform wall thickness—may be an alternative approach. To this end, a 3D dark blood MRI protocol is able to provide the necessary image basis for building models with non-uniform wall thickness^36^.

Third, potential effects of cardiac motion on aortic hemodynamics cannot be assessed with our setup, as there was no contracting left ventricle and/or moving aortic valve. While PWV analyses were focused on the descending aorta and thus are expected to not be impacted, hemodynamics in the ascending aorta may change.

Fourth, the study design did not assess the effects of heart rate or pressure variations on PWV, which remains a controversial topic according to other previous studies. A pre-clinical study with rats reported a positive HR to PWV correlation, which was further pronounced at higher mean arterial pressures^37^. Clinical studies that paced patients at different heart rates found either a positive HR to PWV correlation^38–41^ or no correlation^42,43^. If heart rate to PWV dependencies were to be investigated with the presented in vitro setup, careful considerations need to be made on how to modify the inlet flow rate waveform and whether or not pressures should be regulated with programmed HR changes. Given this open research question, we consider the present flow circuit setup with compliant aorta models to be of high value to further investigate heart rate and pressure variations.

In conclusion, this work demonstrated 3D-printed subject-specific compliant models of the thoracic aorta integrated into a highly-controlled physiological flow circuit for assessment via in vitro MRI. Using compliant rather than rigid models of the aorta is essential to produce realistic flow dynamics and conditions that recapitulate in vivo hemodynamics.

## Supplementary Information

Supplementary Figure S1.

Supplementary Video S2.

Supplementary Video S3.

Supplementary Video S4.

Supplementary Video S5.

Supplementary Information.

## Data Availability

The subject-specific thoracic aorta model and custom-build model-specific connectors (.stl files), as well as all acquired MRI DICOM data is publicly available: https://purl.stanford.edu/dz488kx6180.
