# Supplementary figures and images for: On the impact of vessel wall stiffness on quantitative flow dynamics in a synthetic model of the thoracic aorta
Source: Sci Rep. 2021 Mar 23;11:6703. doi: 10.1038/s41598-021-86174-6 (PMC7988183; doi:10.1038/s41598-021-86174-6)

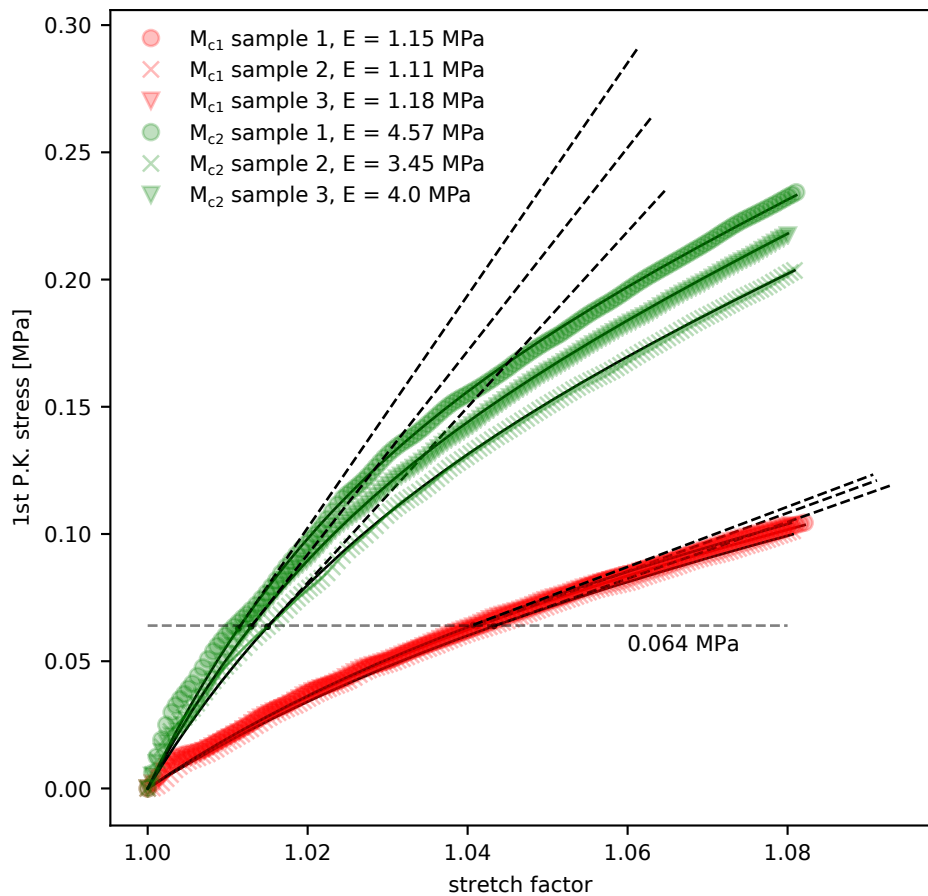

Supplement: Supplementary file 1 — Supplementary Figure S1. [file 41598_2021_86174_MOESM1_ESM.pdf]
