# Supplementary material for: On the impact of vessel wall stiffness on quantitative flow dynamics in a synthetic model of the thoracic aorta
Source: Sci Rep. 2021 Mar 23;11:6703. doi: 10.1038/s41598-021-86174-6 (PMC7988183; doi:10.1038/s41598-021-86174-6)
Supplement: Supplementary file 6 — Supplementary Information. [file 41598_2021_86174_MOESM6_ESM.docx]

**Manuscript title:** On the Impact of Vessel Wall Stiffness on Quantitative Flow Dynamics in a Synthetic Model of the Thoracic Aorta

**Full author list:** Judith Zimmermann*, Michael Loecher, Fikunwa O. Kolawole, Kathrin Bäumler, Kyle Gifford, Seraina A. Dual, Marc Levenston, Alison L. Marsden, Daniel B. Ennis

*Corresponding author: Judith Zimmermann, Lucas MRI Center, 1201 Welch Road, Stanford, CA 94305, USA; E-mail: juzim@stanford.edu.

Supplementary information (Figure/Video legends):

**S1. Tensile testing.** Stress strain data points for the compliant materials of models M_c1_ (red) and M_c2_ (green), each evaluated with three samples. Two-term exponential models were fitted (solid black line). The slope of the tangent lines (dashed) at 1^st^ P.K. stress of 0.053 MPa represent the incremental Young’s moduli (*E_t_*) for each sample. Respective *E_t_* values are given in the legend. Plot created using Python (v3.6, https://www.python.org/).

**S2. Aortic wall expansion.** 2D-cine-GRE data at four landmarks (AAo, BCT, LSA, DAo) for models M_c1_ (red), M_c2_ (green), and M_r_ (blue). Contours were manually delineated in the first frame, then automatically propagated through the cycle. Wall expansion in systole is visible for the compliant models M_c1_ and M_c2_. No periodic wall deformation was visible for model M_r_.

**S3. Particle tracing.** Particle tracing in models M_c1_. Graphic created using MevisLab (v3.4a, https://www.mevislab.de/)

**S4. Particle tracing.** Particle tracing in models M_c2_. Graphic created using MevisLab (v3.4a, https://www.mevislab.de/)

**S5. Particle tracing.** Particle tracing in models M_r_. Graphic created using MevisLab (v3.4a, https://www.mevislab.de/)
